# Conceptualisations of landscape differ across European languages

**DOI:** 10.1371/journal.pone.0239858

**Published:** 2020-10-14

**Authors:** Saskia van Putten, Carolyn O’Meara, Flurina Wartmann, Joanne Yager, Julia Villette, Claudia Mazzuca, Claudia Bieling, Niclas Burenhult, Ross Purves, Asifa Majid

**Affiliations:** 1 Centre for Language Studies, Radboud University Nijmegen, Nijmegen, The Netherlands; 2 Philological Research Institute, National Autonomous University of Mexico, Mexico City, Mexico; 3 The Swiss Federal Institute for Forest, Snow and Landscape Research (WSL), Birmensdorf, Switzerland; 4 Centre for Languages and Literature, Lund University, Lund, Sweden; 5 Department of Geography, University of Zurich, Zurich, Switzerland; 6 Department of Psychology, University of York, York, United Kingdom; 7 Societal Transition and Agriculture, University of Hohenheim, Stuttgart, Germany; 8 Lund University Humanities Lab, Lund University, Lund, Sweden; 9 University Research Priority Programme Language and Space, University of Zurich, Zurich, Switzerland; University of Birmingham, UNITED KINGDOM

## Abstract

Policies aimed at sustainable landscape management recognise the importance of multiple cultural viewpoints, but the notion of landscape itself is implicitly assumed to be homogeneous across speech communities. We tested this assumption by collecting data about the concept of “landscape” from speakers of seven languages of European origin. Speakers were asked to freely list exemplars to “landscape” (a concrete concept for which the underlying conceptual structure is unclear), “animals” (a concrete and discrete concept) and “body parts” (a concrete concept characterised by segmentation). We found, across languages, participants considered listing landscape terms the hardest task, listed fewest exemplars, had the least number of shared exemplars, and had fewer common co-occurrence pairs (i.e., pairs of exemplars listed adjacently). We also found important differences between languages in the types of exemplars that were cognitively salient and, most importantly, in how the exemplars are connected to each other in semantic networks. Overall, this shows that “landscape” is more weakly structured than other domains, with high variability both within and between languages. This diversity suggests that for sustainable landscape policies to be effective, they need to be better tailored to local conceptualisations.

## 1. Introduction

The European Landscape Convention—a key tool in landscape protection, management and planning—puts people at its heart, defining landscape as “an area, as perceived by people, whose character is the result of the action and interaction of natural and/or human forces” [1]. The convention, and many national policies, recognise the importance of gathering cultural viewpoints, for example, by translating questionnaires into national languages and using participative processes to ensure representation of diverse groups. Such approaches recognise that human-nature relations differ across contexts, and so understandings of “landscape” need to take into account diverse perspectives. More generally, a transdisciplinary approach to nature is key to addressing the challenge of sustainable development [2].

Despite the acknowledged differences in the origins of terms used for “landscape” across European cultures [3], there remains a tacit assumption that the notion of landscape is homogeneous across speech communities, at least in Europe [1]. This in turn implies that speakers of different European languages conceptualise the same sort of relationship between humans and nature when they think and talk about “landscape”. We show here that this is not the case through the lens of language as a proxy for culture.

In an increasingly digitised and interconnected world, language is critical for accessing and processing information. Cross-cultural work often uses translations of English lexicons (e.g., to evaluate scenic quality of landscape objects across countries) implicitly promoting the idea that meanings encoded in a lexicon are universal [4]. Such approaches are becoming more common with the availability of machine translation algorithms based on large-scale text analysis [5–10]. However, by assuming one-to-one translatability of words, these approaches do not capture cross-linguistic variability in word meaning which has been shown to exist for a variety of domains—even when comparing closely related languages [11, 12]. Moreover, machine translation algorithms have also been shown to introduce new biases present in the original corpora, for example assigning gender to nouns in stereotypical ways to languages where gendering is not typical [6, 13]. In the case of landscape, this could mean that specific cultural ways of thinking about landscape are imposed upon other cultures. Assumptions of universality are also at odds with evidence gathered in ethnographic and linguistic studies that point to an astonishing diversity in ways of conceptualising and referring to landscape [14, 15]. Given the obvious importance of these issues for cross-cultural policy development and communication, it is remarkable that no large-scale study of “landscape” has been undertaken across major European languages.

We compared conceptualisations of “landscape” in seven languages of European origin, collecting data from over 400 native speakers using cultural domain analysis and the method of free listing [16, 17]. We take advantage of the notion that concepts do not exist in isolation, and their meanings can be explored through their relationships with other terms. Thus, by asking people to list terms in association to a target word, proximity reveals underlying notions related to a concept. Free listing has been used extensively to explore variation in cultural domains, addressing a wide range of concepts such as parts of the human body [18], countries and sports [17, 19] emotions [20] and, closest to our work, geographic features [17, 21].

If “landscape” has a clear shared meaning for speakers within and across languages, similar sets of terms should be produced across speakers, independent of language. This way, we can establish how coherent and stable the concept of landscape is across European languages against other concrete domains that have different organisational principles. We compared free listing of “landscape” to “animals” (a concrete, discrete concept characterised by taxonomy, organised in semantic memory by use—e.g., pets, farm animals, wild animals) [22] and “body parts” (a concrete concept characterised by segmentation, structured as a partonomy, and organised in semantic memory by spatial contiguity) [23]. According to existing English concreteness norms [24], “landscape” is considered as concrete (*M* = 4.34, on a 5-point scale, with 5 indicating “concrete, experience based”), as “animal” (*M =* 4.61) and “body part” (*M* = 4.47). By comparing free-listing of “landscape” to concepts that have taxonomic versus partonomic structure, we can test whether one or the other organizing principle also applies to “landscape”. More importantly, we ask what the structure of “landscape” is, and whether it is the same across the seven languages studied.

## 2. Methods

### 2.1 Participants

A total of 441 speakers of Dutch (Netherlands), English (UK), French (France), German (Switzerland), Italian (Italy), Spanish (Mexico) and Swedish (Sweden) participated in the study (see Table 1). Data from 12 participants was removed because they did not follow the instructions, took the survey a second time, or appeared not to be native speakers of the target language based on answers to the demographic questions. Participants were recruited from universities and personal networks, and in the UK through the online platform Prolific. Participants were rewarded with online gift vouchers worth the equivalent of €20, awarded to one in four participants (randomly selected), or £5 per participant recruited through Prolific.

**Table 1:** Demographic details of the participants.

Language	Country	N	Age (mean)	Age (range)	% female	% student
English	United Kingdom	64	26	18–62	70	67
Dutch	The Netherlands	56	23	17–66	80	84
German	Switzerland	52	30	19–75	73	69
Swedish	Sweden	51	28	20–70	65	96
French	France	80	25	17–59	45	81
Italian	Italy	58	27	19–35	78	59
Spanish	Mexico	68	29	18–49	56	99

Speakers of seven Indo-European languages are included in the study: four from the Germanic sub-family (English, Dutch, German and Swedish) and three from the Romance sub-family (French, Italian and Spanish). The words for “landscape” in the Germanic languages are all cognates (i.e. can be traced back to a single word in the common ancestor language): English *landscape*, Dutch *landschap*, German *Landschaft* and Swedish *landskap*. The Romance words for “landscape” are also cognates, unrelated to the Germanic forms: French *paysage*, Italian *paesaggio* and Spanish *paisaje*. The selected sample of languages allows us not only to look for differences between individual languages, but also to examine whether differences manifest themselves at a language family level. Six of the languages included in the sample are spoken in Europe and one is spoken in Mexico. Mexican Spanish was included because it is closely related to the other Romance languages but spoken in a very different landscape. This allowed us to examine to what extent free listing patterns are the result of living in a specific landscape and to what extent they reflect linguistic and cultural similarities between groups.

### 2.2 Materials

Participants took part in an online survey. In the first part of the survey, participants were asked to list exemplars for three categories: *the landscape*, *body parts* and *animals*. These were translated to the other languages as follows: Dutch: *het landschap*, *lichaamsdelen*, *dieren*; French: *le paysage*, *parties du corps*, *animaux*; German: *die Landschaft*, *Körperteile*, *Tiere*; Italian: *il paesaggio*, *parti del corpo*, *animali*; Spanish: *el paisaje*, *partes del cuerpo*, *animales*; Swedish: *landskapet*, *kroppsdelar*, *djur*. The words for “landscape” were presented with the definite article in order to prompt participants to list features of the landscape rather than landscape types.

In the second part of the survey, participants were first asked their age, gender, the languages they speak, where they are from and whether they are students. Then they were asked how easy it was to list exemplars for the different domains (animals, body parts, landscape) on a scale of 1–5 (very easy/easy/average/difficult/very difficult). They also answered questions about their outdoor and landscape experience. They were asked how much time they spend pursuing outdoor activities in an average week (none/1-2 hours/3-5 hours/6-8 hours/more than 8 hours). After this, they were asked how often they spend time in 11 different kinds of environment: mountains (higher than 1000m), hills (lower than 1000m), forest, countryside, river, lake, coast, desert, volcano, island, and city park. For each environment type, they could choose between the following answers: every day, at least once a week, at least once a month, at least once a year, at least once every 5 years, have been at least once, never visited. Finally, for the same 11 environment types, participants were asked which of them they had visited in the last 6 months, also including the options ‘other’ (with a text box for further specification) and ‘none of the above’.

### 2.3 Procedure

Ethics permission for this study was granted by the Ethics Assessment Committee Humanities at Radboud University Nijmegen. Before beginning the survey, participants were informed about the purpose of the study and gave informed consent. When starting the survey, participants received the following instruction (in their own language): “In this survey, you will be asked to type all the words you know for different categories. For example, if you see the category “vehicles”, you might respond with “car, train, bus, truck” etc.” After this, participants responded to a practice category (fruits) to get familiar with the procedure. We did not examine this data. After the practice category, participants received the following instructions: “There are 3 more categories coming up. For each category, you will have 3 minutes to list as many examples as you can.” Then the three categories, “the landscape”, “body parts” and “animals” appeared one after the other, in random order. For each category, the term appeared at the top of the screen without any further instructions. A counter was also displayed, counting down from three minutes. Participants typed their responses into separate text boxes for each exemplar.

The data was pre-processed in the following manner: all upper-case letters were changed to lower case to allow comparison of strings (note for example that in German all nouns are capitalized, but that typically in tasks such as free listing this capitalization may be neglected). Obvious spelling mistakes and typos were corrected. Alternative spellings of the same word were unified, as well as singular and plural forms of the same word, and the same word with and without articles.

## 3. Results

### 3.1 Listing difficulty

Participants were asked to rate on a scale of 1–5 how difficult it was to list exemplars of the different domains, with 5 indicating very difficult. Across languages participants found listing landscape terms the hardest task. The overall rating for “landscape” was *M* = 3.3, “body parts” *M* = 2.0, and “animals” *M* = 1.6. The data was analysed using a mixed ANOVA with domain as a within-subjects factor (landscape, animals, body parts) and language as a between-subjects factor. We found a significant main effect of domain on people’s self-reported difficulty of listing, *F*(1.83, 770) = 578, *p* < .001, *η*_*p*_^*2*^ = .578. Planned contrasts show that “landscape” was rated as more difficult than both “animals”, *F*(1,422) = 961, *p* < .001, *η*_*p*_^*2*^ = .695, and “body parts”, *F*(1,422) = 508, *p* < .001, *η*_*p*_^*2*^ = .546. There was also a main effect of language, *F*(6, 422) = 3.08, *p* = .006, *η*_*p*_^*2*^ = .042, with pairwise comparisons showing only one significant difference, i.e., the Dutch (*M* = 2.5) found the task harder overall than Italians (*M* = 2.1, *p* = .009, Bonferroni correction). Since we were interested in differences between the domains, and had no specific predictions about differences between languages this effect is not relevant to our research question. Critically, there was no interaction between domain and language *F*(11.0, 770) = 1.10, *p* = .355, *η*_*p*_^*2*^ = .008, and “landscape” was rated the most difficult domain in all languages.

### 3.2 Number of exemplars listed

**Fig 1:**
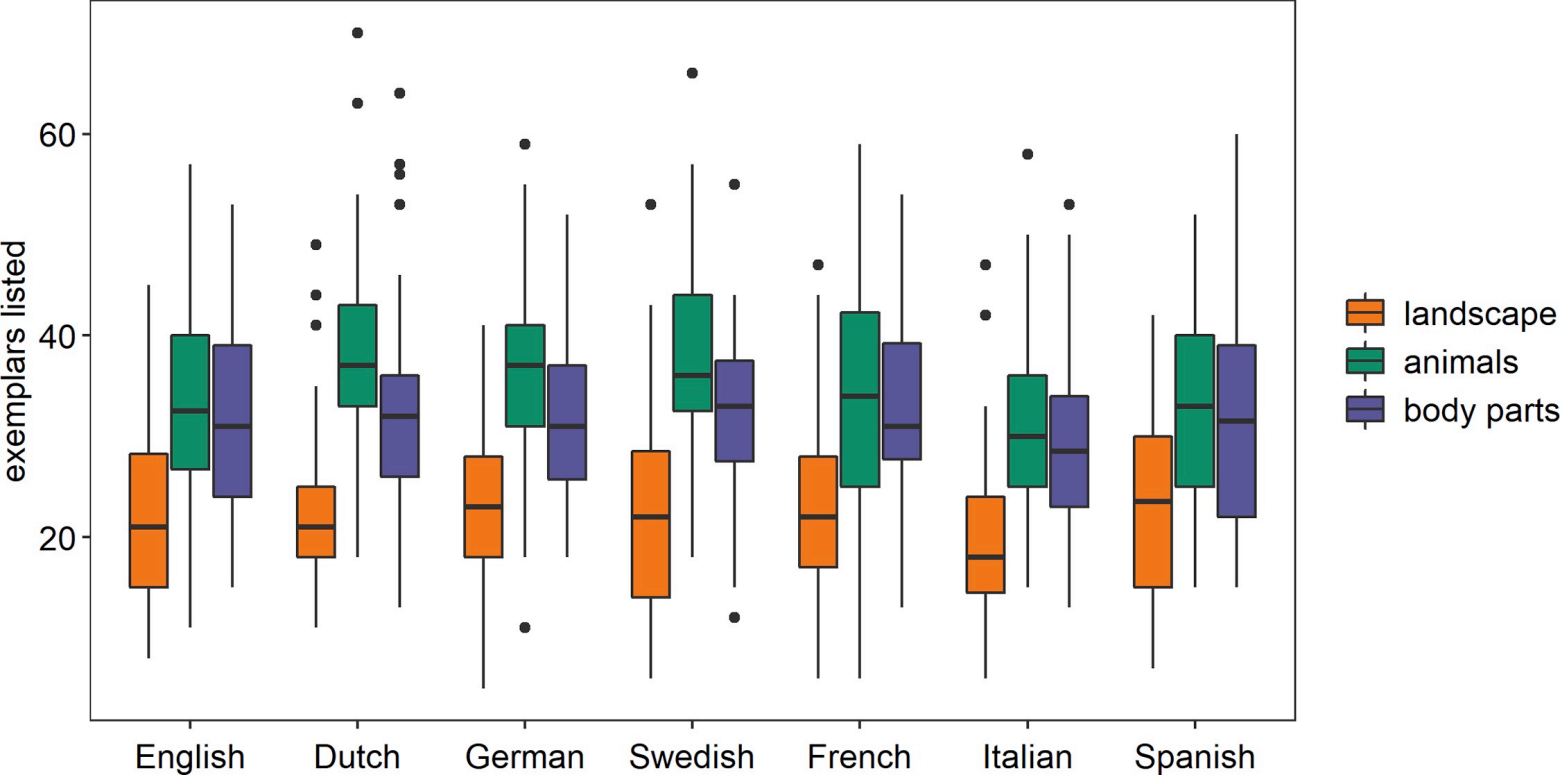
Number of exemplars listed for “landscape”, “animals” and “body parts” by speakers of seven languages. “Landscape” elicited the fewest exemplars in all languages.

Participants also listed fewest exemplars for “landscape”. On average, 22 exemplars were listed for the domain of landscape, 35 for the domain of animals and 32 for the domain of body parts (see Fig 1). The data was again analysed using a 3x7 ANOVA. We found a significant main effect of domain for the number of exemplars listed, *F*(1.96, 826) = 520, *p* < .001, *η*_*p*_^*2*^ = .553. People listed significantly fewer exemplars for landscape than animals, *F*(1,422) = 864, *p* < .001, *η*_*p*_^*2*^ = .672, or body parts, *F*(1,422) = 527, *p* < .001, *η*_*p*_^*2*^ = .556. There is no main effect of language, *F*(6,422) = 1.67, *p* = .127, *η*_*p*_^*2*^ = .023, but there is a significant interaction between language and domain, *F*(11.8, 826) = 2.55, *p* = .003, *η*_*p*_^*2*^ = .035. This was mediated by the relative difference between “animals” and “body parts”. Pairwise comparisons with Bonferroni correction showed that the number of exemplars listed for “animals” is significantly higher than that for “body parts” in Dutch (*p* < .001), English (*p* = .046), German (*p* < .001) and Swedish (*p* < .001), but not in French (*p* = .688), Italian (*p* = .067) and Spanish (*p* = .934). Critically for our research question, landscape exemplars are significantly less numerous than those for both “animals” and “body parts” in all languages (all p-values < .001).

### 3.3 Frequency distributions

**Fig 2:**
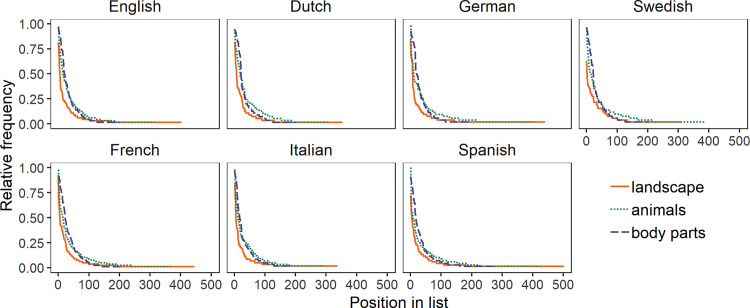
Frequency distributions of exemplars listed for “animals”, “body parts” and “landscape” in seven languages. The x-axis represents an exemplar in an ordered list from most to least frequent. The y-axis represents frequency relative to the number of participants. “Landscape” had fewer high-frequency exemplars than the other domains in all languages, and also more unique exemplars in all but one language.

We next compared the extent to which participants listed the same exemplars within each domain and the same pairs of exemplars adjacently. Fig 2 shows the frequency distributions of exemplars for the three domains across languages. Participants had the least number of shared exemplars for “landscape”. All plots showed the typical Zipfian distribution expected for this type of data, with a few high-frequency exemplars and a long tail of low-frequency exemplars [25]. Typical high-frequency terms were for animals were ‘lion’, ‘tiger’, ‘cat’ and ‘dog’, and for body parts ‘legs’ and ‘arms’.

The landscape distributions initially had steeper slopes than the distributions for the other domains, indicating there were fewer core high-frequency exemplars (more detail to follow). In addition, in all languages except Swedish, the landscape distributions had a longer tail than the distributions for the other domains indicating: (i) there were more low frequency exemplars listed for “landscape” (i.e., terms that were mentioned only once or twice), and (ii) there are more unique exemplars for “landscape” indicating greater differences between people in what was listed in this domain. Swedish deviates from this pattern, most likely due to the meaning of the Swedish word *landskap*, which also refers to an administrative area similar to a province. Out of the 51 Swedish participants, 16 listed place names referring to these provinces. Given that there are a limited number of provinces to choose from, this directly affected the total number of unique terms produced for participants who interpreted *landskap* in this way. Nevertheless, across all languages we see the same distribution across domains showing that “landscape” has a weaker core meaning.

**Fig 3:**
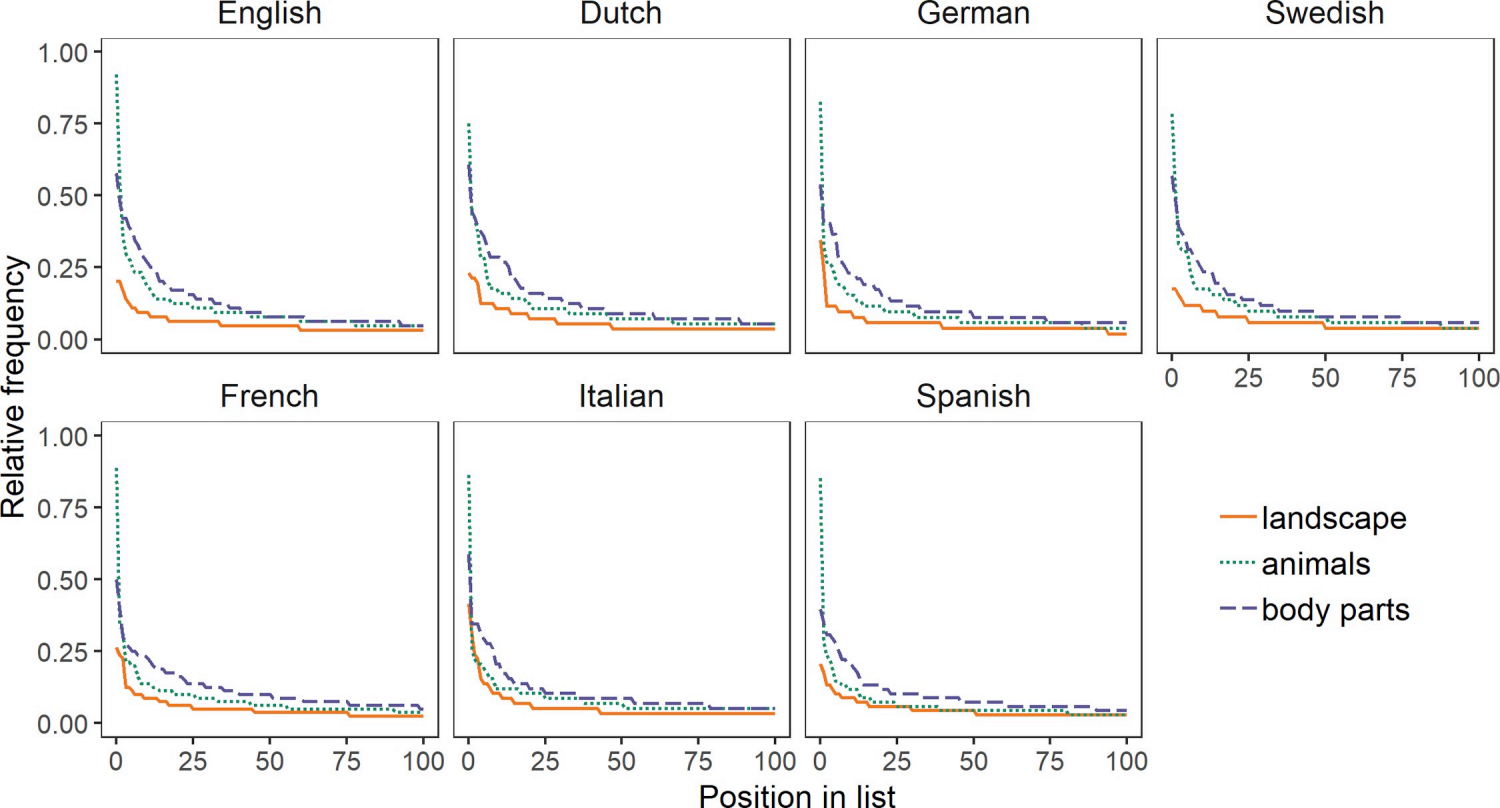
Frequency distributions of co-occurrence pairs for “landscape”, “animals”, and “body parts” in seven languages. The x-axis represents the 100 most frequent co-occurrence pairs ordered from most to least frequent; the y-axis represents frequency relative to the number of participants. In all languages, “landscape” had the fewest common co-occurrence pairs.

Fig 3 depicts the frequencies of co-occurrence pairs for each domain in each language. Co-occurrence pairs are pairs of exemplars listed adjacently in individual lists (e.g. *cat*–*dog* or *hill*–*mountain*). “Landscape” had fewer shared co-occurrence pairs than the other domains in all languages, again indicating more variability in this domain.

### 3.4 Cognitive salience

Next, we zoom in on the specific exemplars listed in the different languages. We calculated the cognitive salience score for each exemplar listed in the three domains. Cognitive salience is a commonly used measure for free listing data, which incorporates both the frequency of an exemplar and its mean rank across all participants, thus giving a measure that takes into account where terms are typically listed in a list [16, 26, 27]. This enables us to compare the salience of exemplars between lists generated by participants. We calculated cognitive salience using the formula *S = F/(NR)* where *F* is frequency, *N* the number of participants, and *R* the mean rank [27]. Tables 2–4 show the five most cognitively salient exemplars for each language for the three domains. Table 2 illustrates that while there are some recurrent salient terms for “landscape”, most notably ‘mountain’, there are also many differences between the languages in which types of landscape elements are most cognitively salient. The most cognitively salient terms for “animals” (Table 3) and “body parts” (Table 4) are less variable between languages: whereas there are 14 unique landscape terms (translation equivalents) between the seven languages, there are only 9 unique body part terms and 8 unique animal terms.

**Table 2:** The five most cognitively salient landscape exemplars in each language.

language	term	translation	cognitive salience	language	term	translation	cognitive salience
English	*hill*		0.15	French	*montagne*	‘mountain’	0.26
	*mountain*		0.14		*mer*	‘sea’	0.10
	*tree*		0.13		*arbre*	‘tree’	0.09
	*grass*		0.09		*forêt*	‘forest’	0.07
	*field*		0.06		*lac*	‘lake’	0.05
Dutch	*gras*	‘grass’	0.16	Italian	*montagna*	‘mountain’	0.25
	*berg*	‘mountain’	0.12		*mare*	‘sea’	0.18
	*boom*	‘tree’	0.11		*collina*	‘hill’	0.14
	*zee*	‘sea’	0.07		*albero*	‘tree’	0.09
	*heuvel*	‘hill’	0.06		*lago*	‘lake’	0.06
German	*Berg*	‘mountain’	0.14	Spanish	*montaña*	‘mountain’	0.16
	*Wiese*	‘meadow’	0.10		*árbol*	‘tree’	0.13
	*Baum*	‘tree’	0.10		*cielo*	‘sky’	0.09
	*Hügel*	‘hill’	0.10		*nubes*	‘clouds’	0.08
	*See*	‘lake’	0.07		*río*	‘river’	0.06
Swedish	*träd*	‘tree’	0.09				
	*Skåne*	(province)	0.07				
	*skog*	‘forest’	0.06				
	*Småland*	(province)	0.05				
	*berg*	‘mountain’	0.05				

**Table 3:** The five most cognitively salient animal exemplars in each language.

language	term	translation	cognitive salience	language	term	translation	cognitive salience
English	*dog*		0.24	French	*chien*	‘dog’	0.27
	*cat*		0.19		*chat*	‘cat’	0.24
	*cow*		0.07		*cheval*	‘horse’	0.06
	*lion*		0.06		*vache*	‘cow’	0.06
	*horse*		0.06		*lion*	‘lion’	0.06
Dutch	*hond*	‘dog’	0.19	Italian	*cane*	‘dog’	0.40
	*kat*	‘cat’	0.15		*gatto*	‘cat’	0.32
	*konijn*	‘rabbit’	0.07		*leone*	‘lion’	0.08
	*paard*	‘horse’	0.07		*topo*	‘mouse’	0.07
	*koe*	‘cow’	0.06		*mucca*	‘cow’	0.05
German	*hund*	‘dog’	0.19	Spanish	*perro*	‘dog’	0.25
	*katze*	‘cat’	0.17		*gato*	‘cat’	0.22
	*maus*	‘mouse’	0.07		*león*	‘lion’	0.06
	*pferd*	‘horse’	0.07		*vaca*	‘cow’	0.04
	*tiger*	‘tiger’	0.06		*tigre*	‘tiger’	0.04
Swedish	*katt*	‘cat’	0.25				
	*hund*	‘dog’	0.21				
	*häst*	‘horse’	0.06				
	*lejon*	‘lion’	0.05				
	*ko*	‘cow’	0.05				

**Table 4:** The five most cognitively salient body part exemplars in each language.

language	term	translation	cognitive salience	language	term	translation	cognitive salience
English	*arm*		0.24	French	*bras*	‘arm’	0.14
	*head*		0.17		*tête*	‘head’	0.12
	*leg*		0.12		*main*	‘hand’	0.10
	*eye*		0.10		*jambe*	‘leg’	0.09
	*finger*		0.09		*nez*	‘nose’	0.08
Dutch	*arm*	‘arm’	0.24	Italian	*mano*	‘head’	0.20
	*been*	‘leg’	0.21		*braccio*	‘arm’	0.15
	*vinger*	‘finger’	0.10		*gamba*	‘leg’	0.11
	*voet*	‘foot’	0.09		*piede*	‘foot’	0.11
	*teen*	‘toe’	0.09		*naso*	‘nose’	0.09
German	*arm*	‘arm’	0.27	Spanish	*cabeza*	‘head’	0.20
	*kopf*	‘head’	0.13		*brazo*	‘arm’	0.12
	*bein*	‘leg’	0.13		*mano*	‘hand’	0.11
	*hand*	‘hand’	0.10		*ojos*	‘eyes’	0.10
	*fuss*	‘foot’	0.09		*nariz*	‘nose’	0.09
Swedish	*arm*	‘arm’	0.22				
	*ben*	‘leg’	0.15				
	*huvud*	‘head’	0.14				
	*finger*	‘finger’	0.09				
	*fot*	‘foot’	0.08				

### 3.5 Semantic networks

To move beyond the small number of exemplars illustrated in Table 2, and visualise how the domains are structured in each of the languages, we created semantic networks [28]. To do so, we first built a co-occurrence matrix for each language, containing counts of all pairs of exemplars that were listed adjacently in the individual free lists. This matrix was used directly as an input to Gephi [29], which was used to create and visualise an undirected graph (meaning that *mountain*–*hill* and *hill*–*mountain* contribute equally to the connectivity of the network), where all exemplars are visualised as nodes, and connected to other nodes with which they co-occurred. We used Louvain community detection [30] as implemented in Gephi to identify groups of related exemplars, as captured by their co-occurrence.

**Fig 4:**
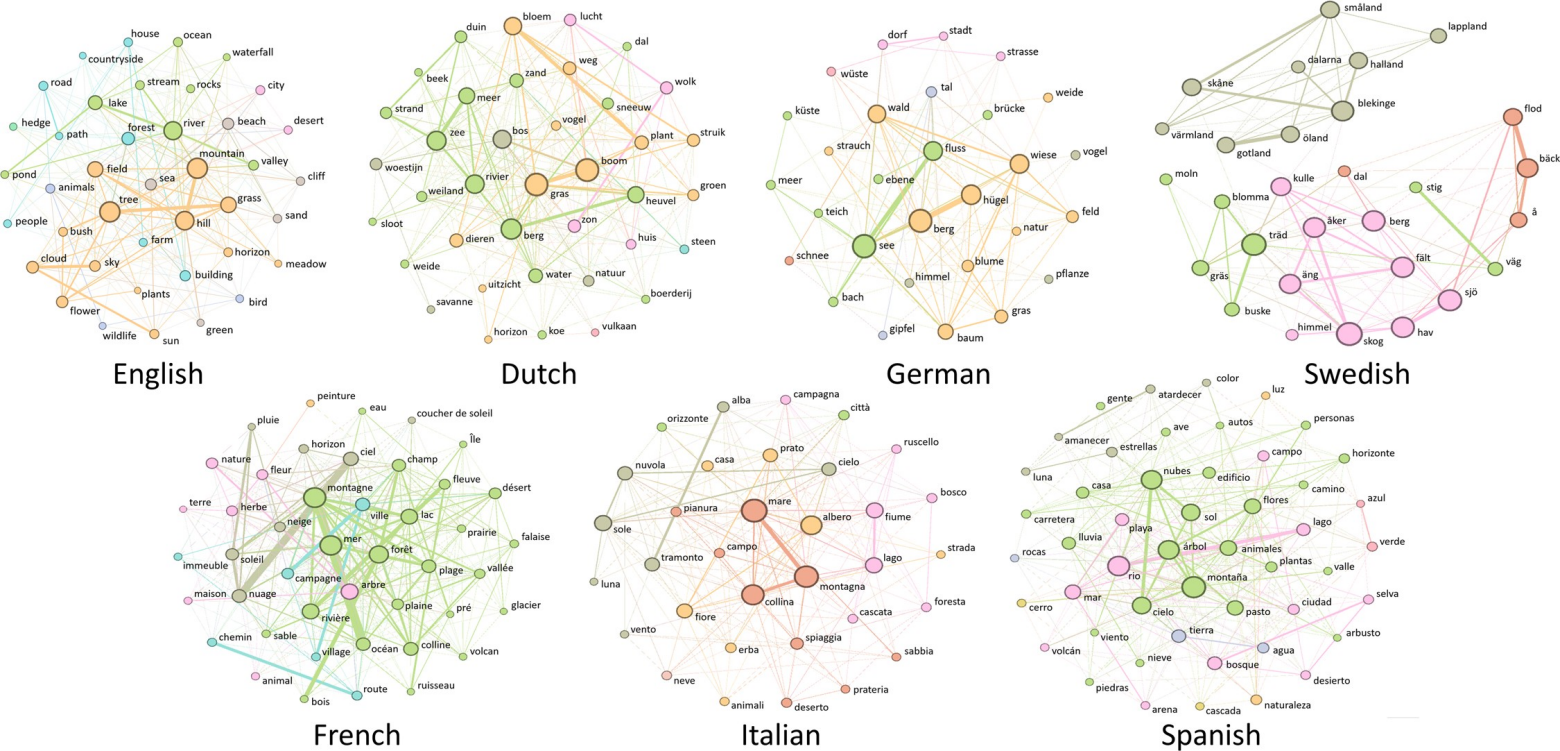
Core landscape concepts across seven languages. Nodes indicate terms, links indicate co-occurrence, and colours indicate clusters within each language.

**Fig 5:**
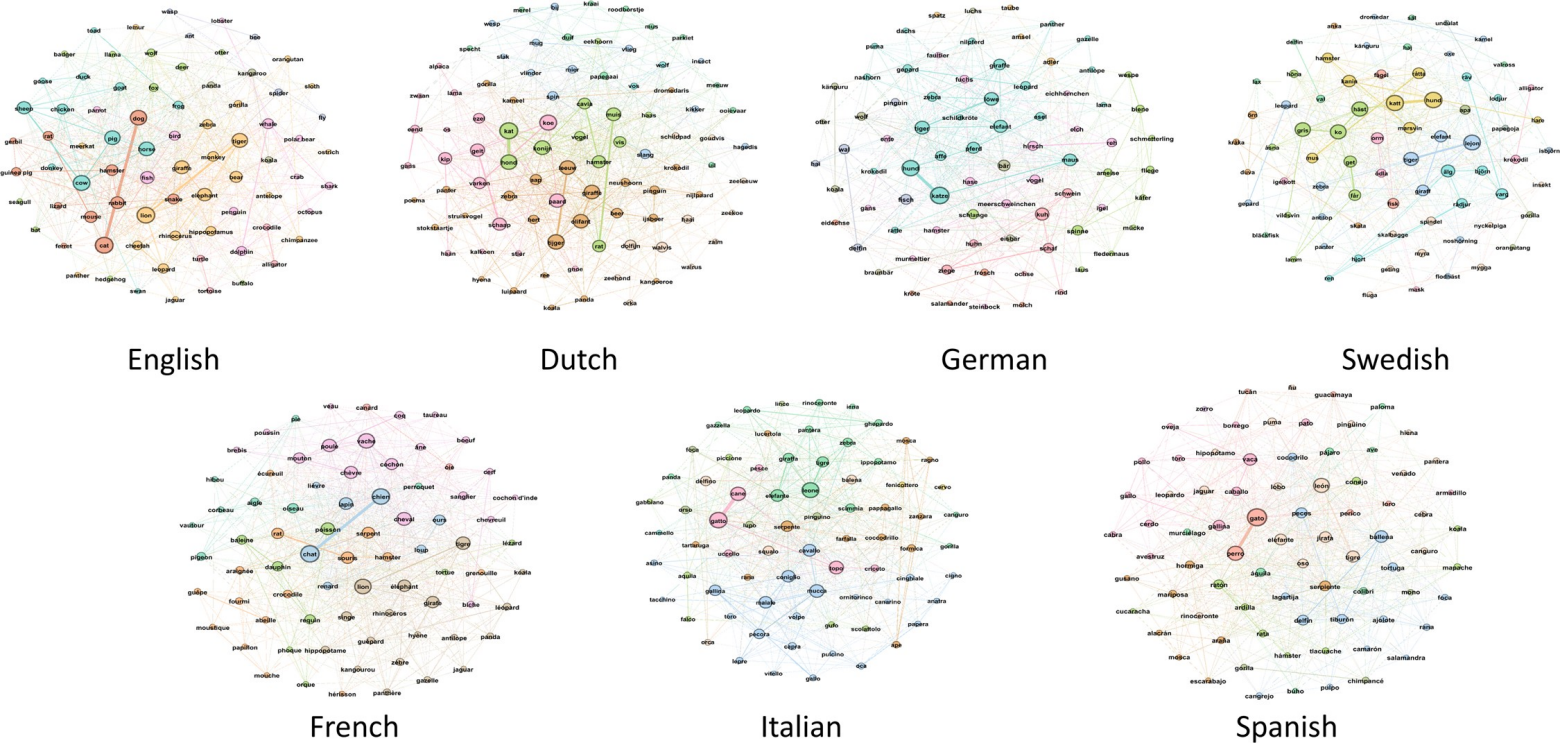
Core animal concepts across seven languages. Nodes indicate terms, links indicate co-occurrence, and colours indicate clusters within each language.

**Fig 6:**
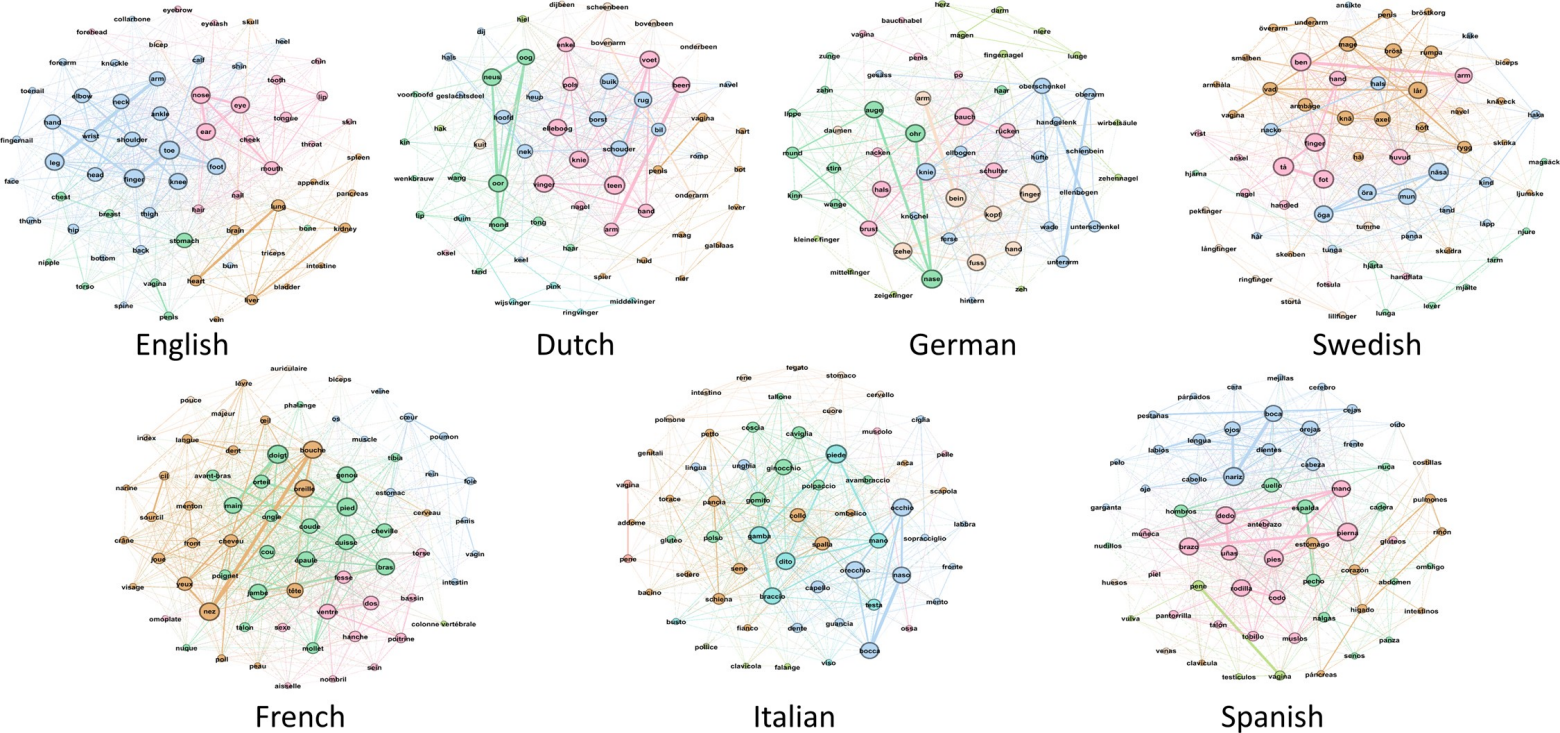
Core body part concepts across seven languages. Nodes indicate terms, links indicate co-occurrence, and colours indicate clusters within each language.

All modularity values were greater than zero, indicating that clustering was non-random ranging from 0.366 for German body parts to 0.548 for Swedish landscape, where two clearly distinct clusters formed due to the polysemy of the term *landskap* in Swedish, as discussed above. We visualised the resulting networks by assigning colours according to the emergent communities (note that colours are assigned independently in each language and do not represent similarities between languages), and weighting node radius and edge widths as a function of weighted degree. Finally, we arranged the resulting networks using the Fruchterman Reingold force-directed layout algorithm. Since we wished to explore the semantic core of the domains in different languages, we filtered nodes of the resulting network according to their weighted degree (i.e., the total number of co-occurrences associated with an individual node). For English we removed all nodes with a weighted degree of less than 15 (equivalent to 23.4% of participants) from the network. For all other languages we filtered using the same proportion, with thresholds for weighted degree used for filtering thus varying between 12 (Swedish) and 19 (French). The resulting networks are shown in Figs 4–6. We see diversity in the nodes (both number and type), density of connections, and number of clusters. All of this together suggests the underlying concept of landscape across languages differs substantially.

To compare the networks between domains, we calculated the average weighted degree and modularity of each of the networks. Average weighted degree captures the connectivity of a network, taking into account not only connections between nodes, but also the strength of the connections. Modularity is a measure of clustering in the network, with higher values indicating the network is easier to partition into clusters. The average weighted degrees of the seven languages for each of the domains were used as input to a one-way ANOVA with domain as factor. There was a significant effect of domain on average weighted degree, *F*(2) = 58.2, *p* < .001, *η*_*p*_^*2*^ = .906. Pairwise comparisons with Bonferroni correction show significant differences between all domains (landscape vs. animals *p* = .003, landscape vs. body parts *p* < .001, and animals vs. body parts: *p* = .007). Critically, “landscape” had the lowest mean weighted degree (*M* = 6.52, *SD* = 0.87), followed by “animals” (*M* = 11.0 *SD* = 1.79) and “body parts” (*M* = 17.8 *SD* = 3.22). This suggests landscape is the least well connected domain (i.e., has fewer and weaker connections between listed exemplars).

A one-way ANOVA also showed a significant effect of domain on modularity, *F*(2) = 4.99, *p* = .027, *η*_*p*_^*2*^ = .454. Post-hoc pairwise comparisons with Bonferroni correction do not show significant differences between pairs of domains, likely due to insufficient power, but the data show that modularity was highest for “landscape” (*M* = .446, *SD* = .051), followed by “animals” (*M* = .443, *SD* = .033) and “body parts” (*M* = .398, *SD* = .018). Higher modularity in landscape may have been caused by the polysemy in Swedish.

Overall, these analyses reinforce the conclusion that “landscape” is less well-defined than “animals” or “body parts”.

### 3.6 Individual differences in landscape experience

In order to explore the extent to which differences in experience with different types of landscape and outdoor life in general affect how people conceptualise “landscape”, we carried out several exploratory analyses.

First we examined whether the number of exemplars listed for landscape correlates with (a) the amount of time people spent outdoors in an average week, (b) the number of landscape types people have visited in their lifetime, and (c) the number of landscape types people visit at least once a month. Landscape types came from a pre-selected set of 11 types for which participants answered questions with respect to their frequency of visits (see Section 2.2). There was a small but significant positive association between the number of landscape types ever visited and the number of landscape exemplars listed, *r*_*s*_(426) = .16, *p* < .001, but no significant association between the number of landscape types visited at least monthly and number of exemplars, *r*_*s*_(426) = .07, *p* = .154, or between the time spent outdoors in an average week and number of exemplars, *r*_*s*_(426) = -.002, *p* = .960.

Second, we explored whether people’s experience with specific landscape elements influenced how they ranked terms referring to those elements in their free listing responses. We selected five landscape terms that occurred among the landscape types in the questionnaire (see Section 2.2) and that were also listed relatively frequently: ‘mountain’ (listed by 77% of people overall), ‘hill’ (46%), ‘forest’ (49%), ‘river’ (49%) and ‘lake’ (47%). For the remaining terms, there was insufficient data to conduct further analyses. We tested the correlation between the frequency with which participants reported visiting these environments and participants’ normalised rank order for each term (and their translation equivalents), considering only those participants who listed the terms and considering only the listed terms that were identical to the term probed in the questionnaire (e.g., the Swedish questionnaire contained the word *backar* ‘hills’, but the most frequent term for ‘hill’ in the free-listing data was *kulle*, so *kulle* was not considered in this analysis). There was a small but significant negative correlation between the frequency with which mountains are visited and the rank of the term for ‘mountain’ in the free-listing data, *r*_*s*_(328) = -.21, *p* < .001, and the same held for ‘forest’, *r*_*s*_(208) = -.14, *p* = .042, and ‘lake’, *r*_*s*_(198) = -.18, *p* = .013, indicating that participants who visited these environment types also listed them earlier in their lists. No significant associations between visiting frequency and rank order were found for ‘hill’, *r*_*s*_(193) = -.07, *p* = .335, or ‘river’, *r*_*s*_(207) = 0.10, *p* = 0.143.

Overall, these analyses show evidence that individual experience also contributes to the shaping of the concept of “landscape”.

## 4. Discussion

Participants found listing landscape exemplars the hardest task, listed fewest of them, had the least number of shared exemplars and shared co-occurrence pairs, and the widest variety of terms among the most cognitively salient exemplars. All of this together indicates that “landscape” is more weakly structured than other domains: it has a smaller core of common exemplars and more variability between speakers in how the individual exemplars are related to each other. This weak structuring is important, since it suggests that simply assuming that speakers will share a common understanding of what is being referred to when we talk about landscape may be incorrect. We find that the notion of “landscape” is particularly variable across languages, suggesting it poses an additional challenge for cross-cultural communication.

Across languages, we find some recurring motifs with respect to what people list under “landscape”. These included exemplars related to landforms (e.g., *mountain*, *hill*), water features (e.g., *river*, *lake*, *sea*), celestial entities (e.g., *sky*, *clouds*, *sun*), land cover (e.g., *grass*, *flowers*, *trees*), and animals (e.g., *birds*, *cows*), suggesting people conceptualise “landscape” in a vague but nonetheless holistic fashion.

The semantic networks (Fig 4) also reveal clear qualitative differences between languages in the conceptualisation of “landscape”. As one way of illustrating these differences, we took cognitively salient terms (Table 2) and explored their co-occurrences to explore semantically related elements within and between languages. For example, for English speakers the most cognitively salient term for “landscape” is *hill* from which we reach *mountain*, *field*, *grass*, *flower*, *green*, *tree*, *beach* and *river*. By contrast, the most cognitively salient term in French, *montagne* ‘mountain’, is related to *mer* ‘sea’, *lac* ‘lake’, *plage* ‘beach’, *riviere* ‘river’, *colline* ‘hill’, *soleil* ‘sun’, *plaine* ‘plain’, *vallée* ‘valley’, *campagne* ‘countryside’ and *forêt* ‘forest’. Critically, French more commonly associates water-related elements to “landscape” through *montagne* despite a similar onset (*montagne* and *hill*).

Differences between the Romance and Germanic language families also emerged from the networks. For example, in the three Romance languages, celestial terms equivalent to, e.g., *sky* and *cloud* were more frequent and central in the networks than in the Germanic languages, where these terms were peripheral or absent. A difference along geographic~cultural rather than linguistic lines was evident in the strong link between ‘mountain’ and ‘sea’ in French and Italian, which was weak or absent in the other languages, pointing to a common Southern European conceptualisation of “landscape”.

The high cognitive salience of ‘mountain’ in all languages (most salient in four languages and among the top five in all; see Table 2) suggests an influence of the general visual prominence of landforms in how “landscape” is conceptualised, and replicates earlier work on frequent geographic categories [17, 21]. However, there are also differences between languages in how strong this influence is: in Swedish and Dutch, the most cognitively salient terms, *träd* ‘tree’ and *gras* ‘grass’ respectively, refer not to salient landforms but rather to land cover. Given the prominence of forests and meadows in the Swedish and Dutch landscapes respectively, this result suggests some influence of the respondents’ environment on their conceptualisation of “landscape”.

More evidence for the influence of people’s environment on their conceptualisation of “landscape” came from the finding that several landscape elements are ranked higher in people’s list if they have visited these elements more frequently. This means that it is likely that individuals’ specific landscape experience has some influence on how they conceptualise landscape. The finding that people who visit a wider range of landscape types each month list more exemplars provides further evidence for the influence of experience. The absence of a correlation between the number of exemplars listed and time spent outdoors more generally or the variety of landscape types ever visited suggests that it is recent and specific experience that matters, not familiarity more generally.

Our results have important implications for policy and research on landscape more generally. First, our study provides a methodology for investigating the concept of “landscape” across communities. We used a well-designed and easily repeatable experiment to gather primary empirical data to explore quantitative and qualitative variation in meaning associated with a domain, and compared this across languages. With increasing efforts to create indicators capable of measuring progress towards global goals [31]—as represented by the United Nations agenda for Sustainable Development, for example—the importance of better understanding how conceptual domains contained within these goals are assigned meaning becomes more imperative. Our study shows one way in which meaning could be studied. The findings could also be used to conduct future more targeted studies using other linguistic and non-linguistic methods, and with different populations.

Second, the holistic but vague conceptualisation of “landscape” implies that policy makers need to be specific in what they mean when they use the term. One approach to reducing the vagueness could be to use cognitively salient terms from the free lists elicited in our experiments in order to specify what aspects of “landscape” are relevant in a specific situation. More broadly, free listing—and related methods—could be used to better measure local understandings so that policies can be better operationalised and evaluated.

Finally, and relatedly, the cross-linguistic variability in the conceptualisation of “landscape” emphasises the importance of taking into account local viewpoints. Political programmes which ignore local conceptualisations are more likely to fail or meet local resistance, and run the risk of ignoring important issues for indigenous and local populations [32]. Since our method not only captures differences in conceptualisations, but points to ways of identifying landscape elements relevant to local conceptualisations (e.g., the emphasis on water found in France) it also suggests a potential way forward, by for example exploring the degree to which policy documents reflect exemplars shared between participants in a given language. Importantly, these exemplars should not simply be translated from an original document, but rather selected from empirically grounded linguistic data related to the domain.

## 5. Conclusion

We found that the concept of landscape is weakly structured and cross-linguistically variable through a free listing task in which speakers of seven languages of European origin were asked to list landscape terms. In fact, landscape showed more variation than other comparable concrete terms, suggesting the challenges for cross-cultural communication are particularly pronounced in this domain. Across languages “landscape” was conceptualised in a holistic fashion, with a small set of core exemplars, but also with notable variation between individuals. Speakers of different languages also conceptualised landscape differently, influenced by distinct language histories, cultures, and geographies. These findings imply that policy makers need to be clear and specific in what they mean when they use the term landscape, and that differences between languages need to be taken seriously when implementing cross-cultural landscape policies.
